# A novel role of LRP5 in tubulointerstitial fibrosis through activating TGF-β/Smad signaling

**DOI:** 10.1038/s41392-020-0142-x

**Published:** 2020-04-29

**Authors:** Xuemin He, Rui Cheng, Chao Huang, Yusuke Takahashi, Yanhui Yang, Siribhinya Benyajati, Yanming Chen, Xin A. Zhang, Jian-xing Ma

**Affiliations:** 1 Department of Endocrinology & Metabolism, The Third Affiliated Hospital of Sun Yat-Sen University, Guangzhou, Guangdong 510630 China; 2 Department of Physiology, College of Medicine, University of Oklahoma Health Sciences Center, Oklahoma City, OK 73104 USA; 3 Harold Hamm Diabetes Center, University of Oklahoma Health Sciences Center, Oklahoma City, OK 73104 USA; 4 NHC Key Laboratory of Hormones and Development (Tianjin Medical University), Tianjin Key Laboratory of Metabolic Diseases, Tianjin Medical University Chu Hsien-I Memorial Hospital & Tianjin Institute of Endocrinology, Tianjin, 300134 China

**Keywords:** Kidney diseases, Kidney diseases

## Abstract

Previous studies by us and others demonstrated that activation of Wnt/β-catenin signaling plays a pathogenic role in chronic kidney diseases (CKD). Wnt co-receptor *LRP5* variants are reported to associate with autosomal dominant polycystic kidney disease; but their exact roles in this disease and renal fibrosis have not been explored. Here, we observed the upregulation of LRP5 in the renal tubules of both type 1 and type 2 diabetic models and of an obstructive nephropathy model. In the obstructed kidneys, *Lrp5* knockout significantly ameliorated tubulointerstitial fibrosis and tubular injury without changing Wnt/β-catenin signaling. Instead, decreased levels of TGF-β1 and TGF-β receptors (TβRs) were detected in *Lrp5* knockout kidneys, followed by attenuated activation and nuclear translocation of Smad2/3 in the renal tubules, suggesting a regulatory effect of LRP5 on TGF-β/Smad signaling. In consistent with this hypothesis, LRP5 overexpression resulted in enhanced TGF-β/Smad signaling activation in renal tubule epithelial cells. Furthermore, LRP5 was co-immunoprecipitated with TβRI and TβRII, and its extracellular domain was essential for interacting with TβRs and for its pro-fibrotic activity. In addition to stabilizing TβRs, LRP5 increased the basal membrane presentation and TGF-β1-induced internalization of these receptors. Notably, TGF-β1 also induced LRP5 internalization. These findings indicate that LRP5 promotes tubulointerstitial fibrosis, at least partially, via direct modulation of TGF-β/Smad signaling, a novel, Wnt-independent function.

## Introduction

Renal fibrosis is a very complex and irreversible pathological process, which involves the activation and crosstalk of a variety of profibrotic signaling pathways,^1^ characterizing the late stages of virtually all types of chronic kidney diseases (CKD). Currently, there are very limited therapeutics to treat renal fibrosis effectively. Aberrant activation of Wnt/β-catenin signaling has been shown to play a pathogenic role in renal fibrosis in obstructive nephropathy^2^ and diabetic nephropathy.^3^ However, the role of low-density lipoprotein receptor-related protein 5 (LRP5), a co-receptor of Wnt/β-catenin signaling,^4^ has not been well characterized in these diseases. A recent study reported that high LRP5 levels in patients were associated with faster progression of idiopathic pulmonary fibrosis (IPF) through activation of Wnt/β-catenin signaling,^5^ implicating an important role of LRP5 in Wnt/β-catenin signaling-related pulmonary fibrotic diseases.

The TGF-β/Smad signaling pathway is a potent fibrogenic pathway. Activation of TGF-β/Smad signaling leads to extracellular matrix synthesis and deposition, podocyte depletion, mesangial expansion, tubule epithelial profibrotic transformation, and myofibroblast activation.^6^ In the kidneys with unilateral ureteral obstruction (UUO), levels of TGF-β receptor I and II (TβRI and TβRII) are predominantly elevated in renal tubules.^7^ Overexpression of a constitutively active mutant of TβRI in mouse renal tubules leads to increases of oxidative stress and inflammatory cell infiltration in the kidney, recapitulating the phenotypes of renal fibrosis.^8^ These studies demonstrate renal tubules as the major action site of TGF-β/Smad signaling in renal fibrosis.

Renal tubules are the primary insult targets in a variety of acute kidney injuries and CKD, and they also play a role in the initiation and progression of renal diseases,^9^ as injured tubule epithelial cells are capable of producing reactive oxygen species,^10^ growth factors,^1,11,12^ chemokines,^1,12^ adhesion molecules,^1,12^ fibrotic cytokines,^1,11,12^ and extracellular matrix.^1,12^ Renal tubule epithelial cells arrested in the prolonged G2/M phase manifest profibrotic effects by expressing fibroblast/myofibroblast markers such as vimentin and alpha-smooth muscle actin (α-SMA).^11^ In addition, tubule epithelial cells interact with multiple cell types to trigger and promote the inflammatory and fibrotic processes.^1^ Notably, profibrotic phenotypes of epithelial cells are positively correlated with the fibrotic severity in human renal allografts.^13^ In type 2 diabetic nephropathy, tubulointerstitial lesions also correlate better with renal dysfunctions than glomerulopathy.^14^ Taking together, these observations suggest a vital role of tubules in contributing to kidney diseases.

Although the expression of LRP5 is detected in normal human and mouse kidneys,^15^ its cellular distribution in the kidney has not been well characterized. Moreover, *LRP5* variants are reported to associate with autosomal dominant polycystic kidney disease,^16^ but the role of LRP5 in renal fibrosis and CKD has not been documented. The present study investigated pro-fibrotic effect of LRP5 in CKD models such as diabetes and UUO, and identified a new role of LRP5 in regulating the TGF-β/Smad signaling pathway to promote renal fibrosis.

## Results

### LRP5 is significantly upregulated in the renal tubules of diabetic and obstructive nephropathies

**Fig. 1:**
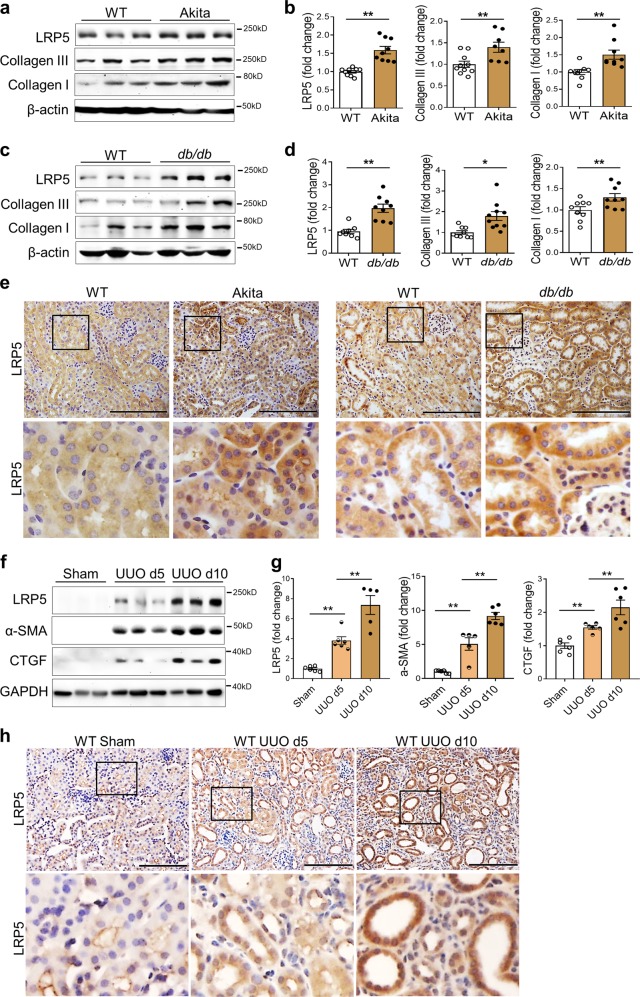
Levels of LRP5 are upregulated in the renal tubules of CKD. **a**, **c** Western blot analyses and (**b**, **d**) densitometry quantification of LRP5, collagen III, and collagen I in the kidney homogenates from 3-month-old Akita and 6-month-old *db/db* mice, as well as their respective age- and genetic background-matched, non-diabetic controls (*n* = 8–10). **e** Immunohistochemical staining of LRP5 (brown color) in the kidneys of Akita and *db/db* mice and their respective non-diabetic controls (*n* = 5–6; scale bar = 100 μm). The lower panels are enlarged images of the boxed areas in the upper panels. **f** Western blot analyses and (**g**) densitometry quantification of LRP5, α-SMA, and CTGF in the kidney homogenates from WT mice with sham surgery or UUO at day 5 and day 10 post-surgery (*n* = 5–6). **h** Immunohistochemical staining of LRP5 (brown color) in the kidneys from WT mice with sham surgery or UUO at day 5 and day 10 (*n* = 5; scale bar = 75 μm). The lower panels are enlarged images of the boxed areas in the upper panels. Each lane represents an individual mouse. All values are expressed as mean ± SEM. *p < 0.05; **p < 0.01, by unpaired Student’s *t*-test

The expression and distribution of LRP5 in CKD have not been previously characterized. Firstly, we measured renal LRP5 levels in diabetic models. Western blot analysis showed that renal LRP5 levels were significantly increased in 3-month-old Akita mice (type 1 diabetes) and 6-month-old *db/db* mice (type 2 diabetes) relative to their genetic background- and age-matched wild-type (WT) non-diabetic controls, accompanied by increased renal levels of collagen I and collagen III (Fig. 1a–d). To determine the cellular localization of LRP5 in the kidney, we used an *Lrp5* knockout (*Lrp5*^*−/−*^) mouse strain, in which the *LacZ* reporter gene is knocked in the locus of *Lrp5*. As shown by X-gal staining, *Lrp5* was abundantly expressed in the renal tubules and moderately in the glomeruli (Supplementary Fig. S1a). Staining of LRP5 confirmed the abundant distribution of LRP5 in the renal tubules of non-diabetic mice, and revealed that the diabetes-induced increases of LRP5 predominantly occurred in the renal tubules of Akita, *db/db*, and OVE26 (type 1 diabetes) mice (Fig. 1e, Supplementary Fig.S2a).

We further measured renal levels of LRP5 in the UUO model, which is also characterized by significant renal fibrosis.^2^ The LRP5 expression in UUO kidneys was evaluated at day 5 and day 10 post-UUO surgery. LRP5 was significantly and persistently upregulated compared to that in the control kidneys, which correlated with the increases of α-SMA and connective tissue growth factor (CTGF) (Fig. 1f, g). Notably, the increase of LRP5 was predominantly in the dilated tubules (Fig. 1h), the major site for the tubulointerstitial fibrosis.

### Knockout of *Lrp5* alleviates the renal tubulointerstitial fibrosis in CKD

As LRP5 levels were positively correlated with those of fibrotic factors in diabetic and obstructed kidneys (Fig. 1), we further evaluated the role of LRP5 in renal fibrosis using *Lrp5*^*−/−*^ mice. Firstly, *Lrp5*^*−/−*^ mice were examined under normal conditions. *Lrp5*^*−/−*^ kidneys displayed similar levels of CTGF, fibronectin, collagen I, and collagen III relative to those of controls (Supplementary Fig. S3a, b). In addition, the ratio of kidney weight to body weight, 24-h urine volume, urinary albumin excretion rate (UAE), and urinary albumin to creatinine ratio (ACR) of *Lrp5*^*−/−*^ mice were not different from those of WT mice with sham surgery (Supplementary Fig. S2c–f). Periodic acid staining (PAS) also showed no obvious difference in glomerular and tubular structures in *Lrp5*^*−/−*^ kidneys (Supplementary Fig. S3g). These findings suggest that *Lrp5* knockout per se does not alter the renal structure and functions.

**Fig. 2:**
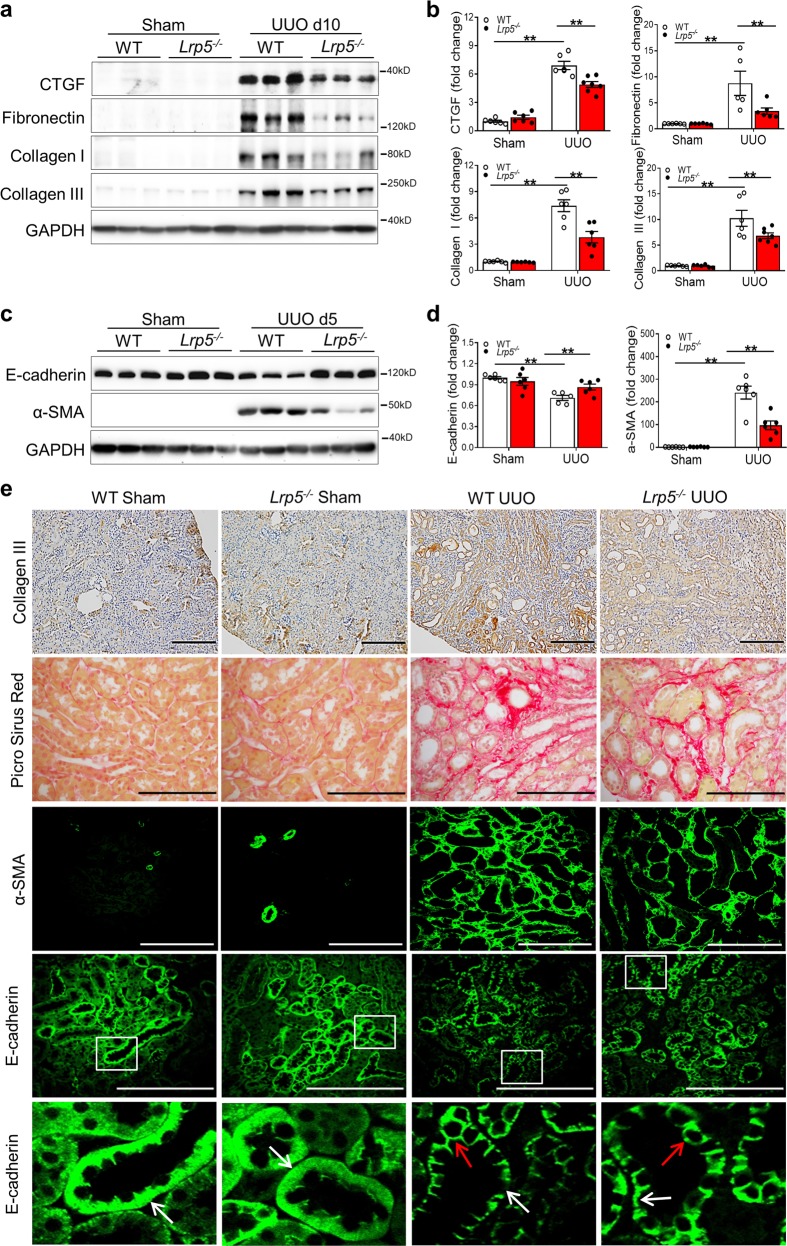
Knockout of *Lrp5* alleviates the renal tubulointerstitial fibrosis in CKD. **a**, **c** Western blot analyses and (**b**, **d**) densitometry quantification of (**a**, **b**) CTGF, fibronectin, collagen I, and collagen III in the kidneys from WT and *Lrp5*^*−/−*^ mice at day 10 post-surgery (*n* = 5–7), and (**c**, **d**) E-cadherin and α-SMA in the kidneys from WT and *Lrp5*^*−/−*^ mice at day 5 post-surgery (*n* = 5–6). **e** Staining of collagen III (brown color; scale bar = 200 μm) and picro-sirius red (red color; scale bar = 200 μm) in the kidneys from WT and *Lrp5*^*−/−*^ mice with sham surgery or UUO at day 10 post-surgery, and staining of α-SMA (green color; scale bar = 75 μm) and E-cadherin (green color; scale bar = 100 μm) in the kidneys from WT and *Lrp5*^*−/−*^ mice with sham surgery or UUO at day 5 post-surgery. The lower panels are enlarged images of the boxed areas in the upper panels. White arrows indicate polarized distribution of E-cadherin in the basolateral membrane of tubules; red arrows indicate re-distribution of E-cadherin to the apical membrane of tubules. *n* = 5–6. Each lane represents an individual mouse. All values are expressed as mean ± SEM. **p < 0.01, by two-way ANOVA with pair-wise multiple comparisons

We utilized UUO to examine the effect of *Lrp5* knockout on renal fibrosis. At day 10 post-UUO surgery, *Lrp5*^*−/−*^ kidneys displayed significantly lower levels of CTGF, fibronectin, collagen I, collagen III, and α-SMA, as shown by Western blot analysis (Fig. 2a, b) and by histochemical staining (Fig. 2e), compared to WT kidneys. In addition, we examined *Lrp5*’s role in renal fibrosis of OVE26 mice, a type 1 diabetic model which displays progressive albuminuria, GFR decline and renal fibrosis,^17^ recapitulating the phenotypes in diabetic patients. In consistent with the observations in the UUO model, *Lrp5* deficiency also resulted in significant reductions of collagen I, collagen III, and α-SMA in the kidneys of OVE26 mice (Supplementary Fig. S2b–d). Due to LRP5’s polarized distribution in renal tubules, we next investigated LRP5’s effect on tubules by measuring the epithelial marker E-cadherin. Notably, UUO not only downregulates E-cadherin expression,^18^ but also induces E-cadherin re-distribution to the apical membrane.^19^ At day 5 post-UUO, *Lrp5* knockout attenuated E-cadherin loss (Fig. 2c, d) and prevented E-cadherin re-distribution (Fig. 2e). Taken together, these results demonstrated that *Lrp5* ablation alleviates renal tubulointerstitial fibrosis.

### Knockout of *Lrp5* attenuates TGF-β/Smad signaling activation in renal tubules

LRP5 is known as a co-receptor of Wnt/β-catenin signaling;^4^ thus, we investigated whether knockout of *Lrp5* affected Wnt/β-catenin signaling in the kidney. Under normal conditions, protein levels of another Wnt co-receptor LRP6 and the effector β-catenin in *Lrp5*^*−/−*^ kidneys were not different from those in WT kidneys (Supplementary Fig. S4a, b). At day 10 post-UUO, although Wnt/β-catenin signaling was activated by UUO, levels of non-phosphorylated β-catenin (non-p-β-catenin) and total β-catenin, key indicators of Wnt/β-catenin signaling activation, were not significantly altered by *Lrp5* knockout (Supplementary Fig. S4c, d). The mRNA levels of Wnt target genes, Cyclin D1^20^ and PPARδ^21^, as well as nine canonical Wnt ligands including Wnt1, Wnt2, Wnt2b, Wnt3, Wnt3a, Wnt8a, Wnt9a, Wnt10b, and Wnt16, were not changed by *Lrp5* knockout (Supplementary Fig. S4e, f). These results suggest that the alleviation of UUO-induced renal fibrosis in *Lrp5*^*−/−*^ mice, at least at day 10 post-UUO, is surprisingly not mediated through the Wnt/β-catenin signaling pathway.

**Fig. 3:**
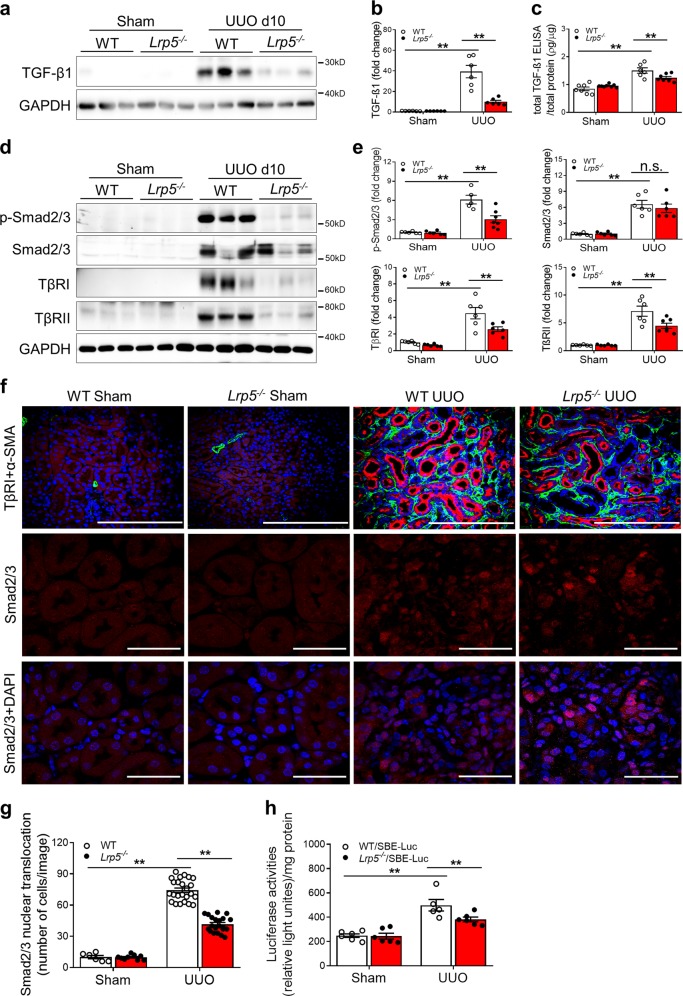
Knockout of *Lrp5* attenuates TGF-β/Smad signaling activation in renal tubules. **a** Western blot analyses, (**b**) densitometry quantification and (**c**) ELISA of total TGF-β1 in the kidneys from WT and *Lrp5*^*−/−*^ mice at day 10 post-surgery (*n* = 6–8). **d** Western blot analyses and (**e**) densitometry quantification of p-Smad2/3, total Smad2/3, TβRI and TβRII in the kidneys from WT and *Lrp5*^*−/−*^ mice at day 10 post-surgery (*n* = 5–7). **f** Immunostaining of TβRI (red color; scale bar = 100 μm) and α-SMA (green color; scale bar = 100 μm) in the kidney sections from WT and *Lrp5*^*−/−*^ mice at day 5 post-surgery (*n* = 5–7), and of Smad2/3 (red color; scale bar = 25 μm), and (**g**) quantification of nuclear Smad2/3 in the kidney sections from WT and *Lrp5*^*−/−*^ mice at day 10 post-surgery (*n* = 5–10). **h** Luciferase activity measurement in the kidney homogenates from WT/SBE-Luc and *Lrp5*^*−/−*^*/*SBE-Luc mice at day 10 post-surgery (*n* = 5–6). Each lane represents an individual mouse. All values are expressed as mean ± SEM. **p < 0.01; n.s., not significant, by two-way ANOVA with pair-wise multiple comparisons

Previously, *Lrp5* deficiency was reported to result in decreased TGF-β1 production in a pulmonary fibrosis model.^5^ Therefore, we examined TGF-β1 levels at day 10 post-UUO. Consistently, total TGF-β1 levels were significantly lower in the *Lrp5*^*−/−*^ UUO kidneys, compared to those in the WT UUO kidneys (Fig. 3a–c). As TGF-β1 is a potent stimulator of TGF-β/Smad signaling,^22^ we measured this signaling pathway. UUO increased the levels of phosphorylated Smad2 and Smad3 (p-Smad2/3), Smad2/3, TβRI, and TβRII; meanwhile, *Lrp5* knockout down-regulated renal levels of these factors in UUO (Fig. 3d, e). Furthermore, we examined TβRI cellular localization at day 5 post-UUO, when the tubular structure was still mostly intact, and the tubulointerstitial fibrosis was prominent. As shown in Fig. 3f, TβRI was predominantly upregulated in the tubules of the UUO kidneys, and not co-localized with α-SMA in the interstitial areas. *Lrp5* knockout attenuated the upregulation of TβRI in the UUO kidneys. The nuclear translocation of Smad2/3 was also substantially decreased in *Lrp5*^*−/−*^ kidneys compared to WT at day 10 post-UUO (Fig. 3f, g). Furthermore, by crossbreeding *Lrp5*^*−/−*^ mice with SBE-Luc mice, a reporter strain for TGF-β/Smad signaling,^23^ we generated an *Lrp5*^*−/−*^*/*SBE-Luc strain. Consistently, *Lrp5* knockout attenuated UUO-induced Smad2/3 activities at day 10 post-UUO (Fig. 3h). Interestingly, the depletion of *Lrp5* in the kidney did not affect basal TGF-β/Smad signaling under normal conditions (Supplementary Fig. S3h, i). Taken together, these results suggest that inhibition of TGF-β/Smad signaling contributes to the ameliorated tubulointerstitial fibrosis in *Lrp5*^*−/−*^ UUO kidneys.

### LRP5 promotes TGF-β/Smad signaling in renal tubule epithelial cells

**Fig. 4:**
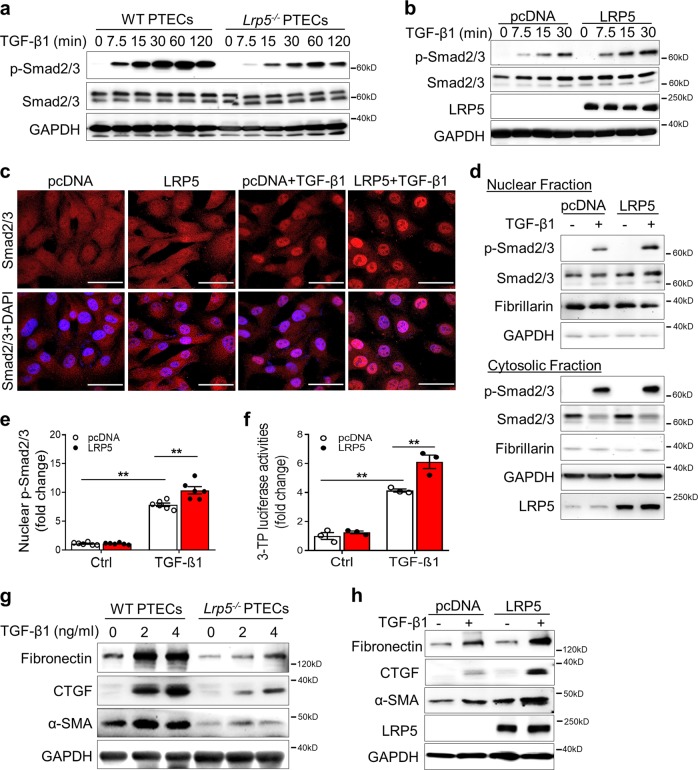
LRP5 promotes TGF-β/Smad signaling in renal tubule epithelial cells. **a**, **b** Western blot analyses of p-Smad2/3 and total Smad2/3 (**a**) in WT PTECs and *Lrp5*^*−/−*^ PTECs treated with 2 ng/ml TGF-β1 for the indicated times, and (**b**) in HKC-8 cells after transfection of a control plasmid (pcDNA3) or a plasmid expressing LRP5 for 48 h, followed by the treatment with/without 2 ng/ml TGF-β1 for the indicated times. **c** Immunostaining of Smad2/3 (red color; scale bar = 25 μm) in HKC-8 cells after transfection of a pcDNA3 plasmid or a plasmid expressing LRP5 for 48 h, followed by the treatment with/without 2 ng/ml TGF-β1 for 60 min. **d** Western blot analyses and (**e**) densitometry quantification of nuclear levels of p-Smad2/3 and Smad2/3 in the fractions extracted from HKC-8 cells after transfection of a pcDNA3 plasmid or a plasmid expressing LRP5 for 48 h, followed by the treatment with/without 2 ng/ml TGF-β1 for 60 min (*n* = 6). **f** 3TP-Lux luciferase activity assay in HKC-8 cells after transfection of the 3TP-Lux plasmid, a renilla plasmid, and a pcDNA3 plasmid or a plasmid expressing LRP5 for 48 hr, followed by the treatment with/without 4 ng/ml TGF-β1 for 16 h. Relative luciferase activity was presented as folds of that in the cells with transfection of pcDNA3 control (*n* = 3). **g**, **h** Western blot analyses of fibronectin, CTGF, and α-SMA (**g**) in primary PTECs from WT and *Lrp5*^*−/−*^ mice exposed to the indicated concentrations of TGF-β1 for 24 h, and (**h**) in HKC-8 cells after transfection of a pcDNA3 plasmid or a plasmid expressing LRP5 for 48 h, followed by the treatment with/without 2 ng/ml TGF-β1 for 24 h. Representative images are shown from at least 3 independent experiments. All values are expressed as mean ± SEM. **p < 0.01, by two-way ANOVA with pair-wise multiple comparisons

Our results demonstrated renal tubules as the major action site of UUO-induced LRP5 and TGF-β/Smad signaling, and renal tubule epithelial cells were thus utilized as a cell model in this study. Primary proximal tubule epithelial cells (PTECs) were isolated and confirmed by immunostaining of sodium-glucose linked transporter 1 (SGLT-1) (Supplementary Fig. S5). In *Lrp5*^*−/−*^ PTECs, TGF-β1-elicited Smad2/3 phosphorylation was attenuated compared to that in WT PTECs (Fig. 4a). On the other hand, overexpression of LRP5 in HKC-8 cells, a human proximal tubule epithelial cell line, potentiated TGF-β1-induced Smad2/3 phosphorylation (Fig. 4b) and nuclear translocation (Fig. 4c). Measurement of nuclear fractions further demonstrated higher nuclear p-Smad2/3 levels in LRP5-overexpressing cells (Fig. 4d, e). As shown by 3TP-Lux luciferase assay, a reporter system of Smad2/3 transcriptional activity,^24^ LRP5 overexpression enhanced the transcriptional activity of Smad2/3 induced by TGF-β1 (Fig. 4f). We also measured the expression of its downstream target genes fibronectin, CTGF, and α-SMA.^25–27^ Consistently, fibronectin, CTGF, and α-SMA expression was enhanced by LRP5 overexpression and attenuated by *Lrp5* knockout (Fig. 4g, h). Thus, we have demonstrated that LRP5 promotes TGF-β/Smad signaling in renal tubule epithelial cells.

### LRP5 interacts with TβRs and promotes the formation of TβRI/TβRII heterodimers

**Fig. 5:**
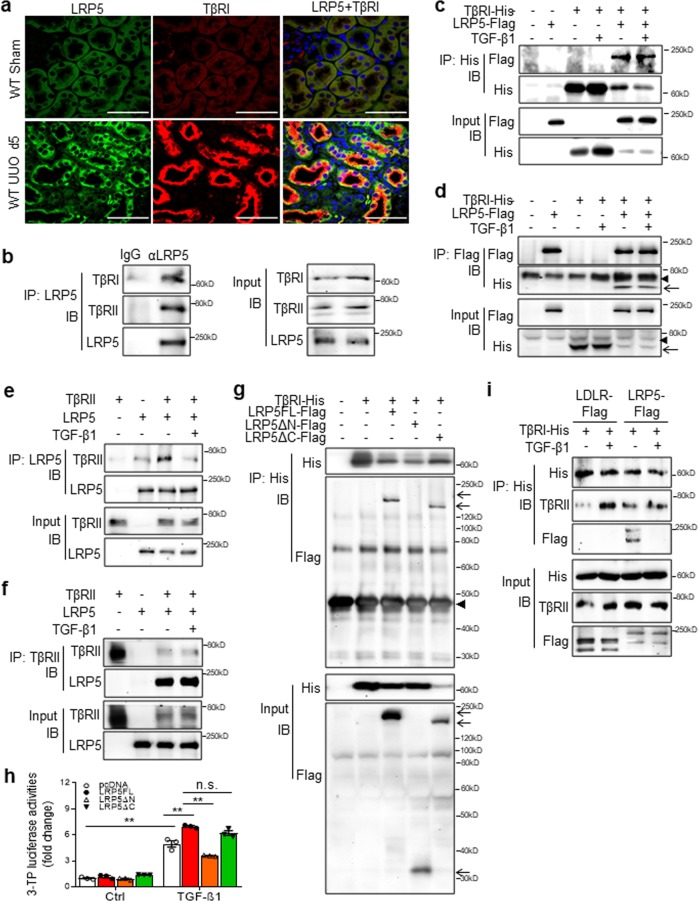
LRP5 interacts with TβRs and stimulates the formation of TβRI/TβRII heterodimers. **a** Immunostaining of LRP5 (green color; scale bar = 25 μm) and TβRI (red color; scale bar = 25 μm) in the kidney sections from WT mice at day 5 post-surgery showing co-localization of LRP5 and TβRI in the injured tubules of UUO kidneys (*n* = 5–7). **b** IP of endogenous LRP5 in HKC-8 cells. LRP5 was precipitated with its specific antibody. The precipitated proteins were immunoblotted for TβRI, TβRII, and LRP5. **c**, **d** IP of (**c**) TβRI and (**d**) LRP5 in HEK293A cells co-transfected with TβRI-His and LRP5-Flag for 48 h, followed by the treatment with/without 2 ng/ml TGF-β1 for 15 min. TβRI-His was precipitated by Ni-NTA resin and LRP5-Flag by anti-Flag resin. The precipitated proteins were immunoblotted for His and Flag. **e**, **f** IP of (**e**) LRP5 or (**f**) TβRII in HKC-8 cells co-transfected with TβRII and LRP5 for 48 h, followed by the treatment with/without 2 ng/ml TGF-β for 15 min. TβRII and LRP5 were precipitated using their specific antibodies. The precipitated proteins were immunoblotted for TβRII and LRP5. **g** IP of TβRI in HEK293A cells co-transfected with TβRI-His and LRP5FL-Flag, LRP5∆N-Flag or LRP5∆C-Flag for 48 h. TβRI-His was precipitated by Ni-NTA resin. The precipitated proteins were immunoblotted for His and Flag. **h** Measurement of 3TP-Lux luciferase activity in HKC-8 cells after transfection of the 3TP-Lux plasmid, a renilla plasmid and a control plasmid or plasmid expressing LRP5FL, LRP5∆N, or LRP5∆C for 48 h, followed by the treatment with/without 4 ng/ml TGF-β1 for 16 h. Relative luciferase activity was presented as folds of that in the cells with transfection of pcDNA3 control (*n* = 3). **i** IP of TβRI in HKC-8 cells co-transfected with TβRI-His and LRP5-Flag or LDLR-Flag for 48 h, followed by the treatment with/without 2 ng/ml TGF-β for 15 min. TβRI-His was precipitated by Ni-NTA resin. The precipitated proteins were immunoblotted for His, Flag, and TβRII. Arrowheads indicate non-specific bands; arrows indicate target proteins. All values are expressed as mean ± SEM. **p < 0.01; n.s., not significant, by two-way ANOVA with pair-wise multiple comparisons

We showed that UUO-induced prominent over-expression of LRP5 and TβRI in renal tubules. To understand whether UUO-induced LRP5 was co-localized with TβRI, we examined the UUO kidneys at day 5. Indeed, LRP5 and TβRI were co-localized in the dilated tubules of UUO kidneys (Fig. 5a). Using co-immunoprecipitation (Co-IP) in normal condition, endogenous LRP5 was found to co-precipitate with both endogenous TβRI and TβRII in renal tubule epithelial cells (Fig. 5b). By forced overexpression of LRP5 and TβRI in 293A cells, LRP5 also interacted with TβRI, which was independent of TGF-β1 (Fig. 5c, d). Similar to TβRI, TβRII was co-precipitated with LRP5, independent of TGF-β1 (Fig. 5e, f). To identify the structural domain of LRP5 responsible for interacting with TβRs, we generated plasmids expressing the full-length LRP5 (LRP5FL), an LRP5 truncation mutant lacking the extracellular domain (LRP5ΔN), or an LRP5 truncation mutant lacking the intracellular domain (LRP5ΔC). LRP5FL and LRP5ΔC, but not LRP5ΔN, were associated with TβRI (Fig. 5g). 3TP-luciferase activity assay further confirmed that only LRP5FL and LRP5ΔC potentiated TGF-β/Smad signaling in HKC-8 cells (Fig. 5h), suggesting that the extracellular domain of LRP5 confers the potentiating effect on TGF-β/Smad signaling.

Next, we evaluated the effect of LRP5 on the formation of TβRI/TβRII heterodimers, an essential step for signaling activation.^28^ In the absence of TGF-β1, TβRI-His failed to co-precipitate with TβRII in the low-density lipoprotein receptor (LDLR) control group, consistent with the report that TβRI is separated from TβRII in quiescent state.^29^ In the LRP5-expressing cells, a significant amount of TβRII co-precipitated with TβRI was detected (Fig. 5i), suggesting that LRP5 alone promotes the formation of TβRI/TβRII heterodimers. Notably, LRP5 was also co-precipitated with TβRI/TβRII heterodimers. Upon TGF-β1 stimulation, TβRI quickly binds to TβRII, forming TβRI/TβRII heterodimers which subsequently activates Smad2/3^28^. Indeed, after TGF-β1 treatment for 15 min, a significant amount of co-precipitated TβRII was detected in the LDLR control group. In the LRP5 group, co-precipitation of TβRII was decreased (Fig. 5i), which is ascribed to the fact that TβRI dissociates from TβRII after Smad2/3 becomes phosphorylated.^30^ Furthermore, we noticed that LRP5 overexpression significantly increased the protein levels but not mRNA levels of TβRs (Supplementary Fig. S6a–c). As shown by protein stability analysis, LRP5 prolonged the half-lives of TβRs (Supplementary Fig. S6d, e).

### LRP5 promotes the basal level membrane presentation and ligand-induced internalization of TβRs

**Fig. 6:**
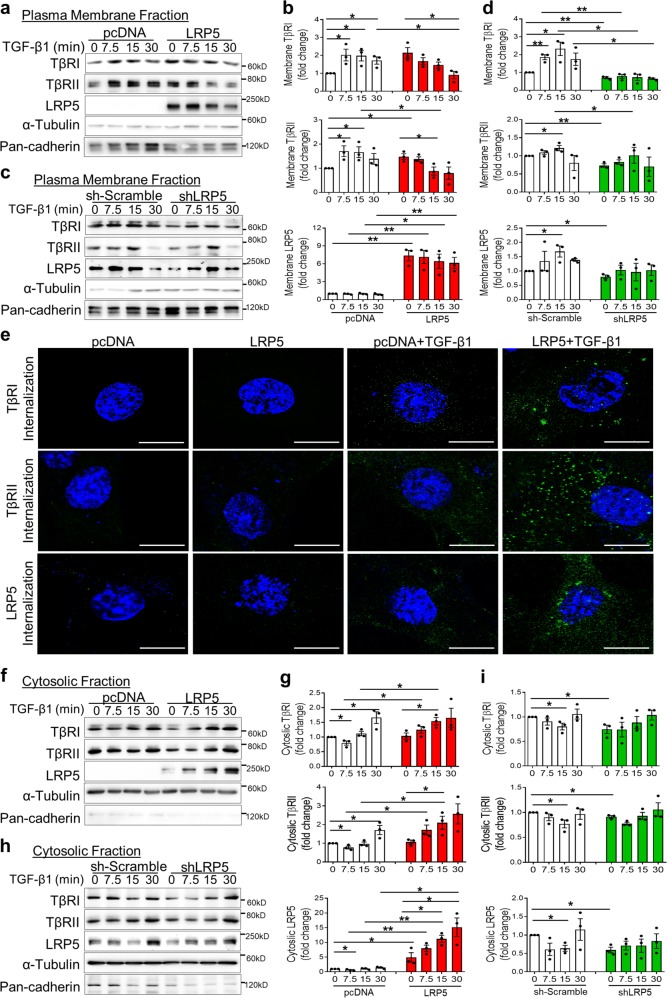
LRP5 promotes the basal membrane presentation and ligand-induced internalization of TβRs. **a**, **c**, **f**, **h** Western blot analyses and (**b**, **d**, **g**, **i**) densitometry quantification of TβRI, TβRII, and LRP5 in the cell factions from HKC-8 cells after transfection with (**a**, **b**, **f**, **g**) a control plasmid (pcDNA3) or a plasmid expressing LRP5, and (**c**, **d**, **h**, **i**) a control plasmid (sh-scramble) or a plasmid expressing shRNA for LRP5 (shLRP5) for 48 h, followed by the treatment of 2 ng/ml TGF-β1 for the indicated times. Cells were fractionated and immunoblotted for TβRI, TβRII, and LRP5, with pan-cadherin and α-tubulin as loading controls (*n* = 3). **e** Immunostaining of internalized TβRI, TβRII, and LRP5 in HKC-8 cells after transfection of pcDNA3 or a plasmid expressing LRP5 together with either a plasmid expressing TβRI or TβRII for 48 hr, followed by the treatment of 2 ng/ml TGF-β1 for 7.5 min (green color; scale bar = 10 μm). Representative images are shown from at least three independent experiments. All values are expressed as mean ± SEM. *p < 0.05; **p < 0.01, by unpaired Student’s *t*-test

The availability of TβRs on the plasma membrane is another important factor affecting TGF-β signaling.^28^ Firstly, LRP5 was detected in the membrane, and its basal membrane levels were ~60% of total LRP5 in endogenous and forced overexpressed conditions (Supplementary Fig. S1b, d, e). Cells were fractionated to examine the dynamics of membrane TβRs in response to TGF-β1 stimulation. Notably, LRP5 overexpression alone increased the basal membrane levels of TβRI and TβRII. Upon TGF-β1 treatment, membrane TβRI and TβRII levels were transiently increased at 7.5–15 min of TGF-β1 treatment in the control pcDNA3 vector group, and then gradually decreased. However, membrane TβRI and TβRII levels were persistently decreased in the LRP5-overexpressing group (Fig. 6a, b). Interestingly, TGF-β1 also decreased the membrane levels of LRP5, correlating with the changes of membrane TβRs (Fig. 6a, b). We also examined the knockdown effect of LRP5 using a short hairpin RNA targeting LRP5 (shLRP5). Contrary to the effect of LRP5-overexpression, basal membrane levels of TβRI and TβRII were significantly decreased by LRP5 knockdown alone, compared to those in the control group. Furthermore, the TGF-β1-induced membrane presentation of its receptors was attenuated by LRP5 knockdown at 7.5–15 min (Fig. 6c, d).

In addition, we examined the dynamics of internalized TβRs by evaluating the cytosolic fractions. In the control group, cytosolic TβRI and TβRII levels were transiently reduced at 7.5–15 min of TGF-β1 treatment, compared to those at 0 min, due to the trafficking of these receptors to the plasma membrane. After 7.5–15 min, cytosolic TβRI and TβRII were gradually increased. With LRP5 overexpression, cytosolic TβRs were continuously increased after TGF-β1 treatment (Fig. 6f, g). Immunostaining of internalized TβRs at 7.5 min also confirmed enhanced internalization of these receptors by LRP5 overexpression (Fig. 6e). Interestingly, we noticed that LRP5 was also internalized by upon TGF-β1 stimulation, correlating with the changes of TβRs. The increases of cytosolic TβRs were unlikely to result from de novo synthesis, as treatment of TGF-β1 for 30 min did not increase the total levels of these receptors (Fig. S7). On the contrary, knockdown of LRP5 induced decreases of cytosolic levels of TβRs (Fig. 6h, i). Taken together, these results suggest that LRP5 potentiates the basal level membrane presentation of TβRs and their internalization elicited by TGF-β1.

## Discussion

The present study identified a previously undocumented pro-fibrotic function of LRP5 through direct interactions with TβRs in renal fibrosis. Notably, LRP5 was upregulated in the renal tubules of CKD models. Moreover, knockout of *Lrp5* ameliorated the tubulointerstitial fibrosis in UUO kidneys, independent of Wnt/β-catenin signaling. The results of Smad2/3 nuclear translocation and luciferase activity in SBE-Luc reporter mice provided solid in vivo evidence that *Lrp5* knockout significantly attenuated UUO-induced TGF-β/Smad signaling activation. On the other hand, overexpression of LRP5 potentiated TGF-β/Smad signaling in renal tubule epithelial cells. As to the mechanism underlying this profibrotic activity of LRP5, the present study showed for the first time that LRP5 interacted directly with TβRI and TβRII, prolonged their half-lives, promoted the formation of TβRI/TβRII heterodimers, and enhanced membrane presentation and TGF-β1-elicited internalization of these receptors. Our studies presented evidence suggesting that the pro-fibrotic activity of LRP5 in the kidney is through a mechanism independent of Wnt/β-catenin signaling, which may represent a new pathogenic mechanism for the progression of renal fibrosis in CKD.

This study showed that LRP5 was upregulated in the renal tubules of both type 1 and type 2 diabetic models (Fig. 1e) and of the UUO model (Fig. 1h), consistent with previous studies.^31,32^ In alignment, we found that both high glucose and TGF-β1 potently increased LRP5 levels in renal tubule epithelial cells in vitro (Supplementary Fig. S8), which recapitulated the changes of LRP5 in diabetic and UUO kidneys, respectively. However, TGF-β1 did not significantly prolong the half-life of LRP5 protein (Fig. S9), suggesting that the increase of LRP5 may be regulated at the transcriptional level. Thus, it remains to be elucidated how TGF-β1 regulates the transcription of LRP5. Notably, diabetes-induced LRP5 was predominantly located on the apical membrane and microvilli of renal tubules, although no obvious polarized distribution of LRP5 was detected in those of non-diabetic kidneys (Supplementary Fig. S1c). We assume that this polarized distribution of LRP5 may play a role in reabsorbing glucose by renal tubules in response to diabetes, a property of LRP5 which has been demonstrated in mammary epithelial cells.^33^

On the other hand, LRP5 affected TGF-β1 levels, as our results showed that renal levels of total TGF-β1 were reduced in *Lrp5*^*−/−*^ UUO kidneys, which was consistent with decreased total TGF-β1 production in IPF.^5^ It is not well understood how LRP5 affects total TGF-β1 levels in these fibrotic diseases. The regulation of TGF-β1 levels by LRP5 is unlikely through Wnt/β-catenin signaling,^12^ as the Wnt/β-catenin signaling activity was not significantly altered by *Lrp5* knockout at day 10 post-UUO (Supplementary Fig. S4c–f). It has been reported that TGF-β/Smad signaling upregulates TGF-β1 expression,^34^ which may explain the regulation of total TGF-β1 by LRP5. Interestingly, *Lrp5* deficiency did not reduce the response to TGF-β1 in lung fibroblasts and alveolar epithelial cells,^5^ which is contrast to our observation in renal tubule epithelial cells (Fig. 4). It is noteworthy that LRP5 expression was not measured in lung fibroblasts or alveolar epithelial cells in the previous study, while LRP5 was abundant in renal tubule epithelial cells. Therefore, it is unclear whether the divergent responses to TGF-β1 are attributed to different expression levels of LRP5 in these cell types.

In the absence of TGF-β1, increased formation of TβRI/TβRII heterodimers was observed after LRP5 overexpression (Fig. 5i), a property which is also possessed by neuropilin-1,^35^ another co-receptor of TGF-β/Smad signaling. It is not known why overexpression of this Wnt co-receptor alone could trigger the formation of TβRI/TβRII heterodimers, which theoretically only occurs when TGF-β1 is present. Notably, when TGF-β1 was applied, LRP5 internalization was detected, sharing the same trend as TβRs, which also indirectly indicates that LRP5 binds to TβRI/TβRII heterodimers to undergo internalization.

It is well-known that the crosstalk between Wnt/β-catenin signaling and TGF-β/Smad signaling occurs at multiple subcellular levels including the extracellular, cytoplasmic, and nuclear levels.^36^ TGF-β/Smad increases the levels of Wnt ligands and enhances Wnt ligand-elicited β-catenin stabilization in vascular smooth muscle cells.^37^ In addition, TGF-β1 suppresses DKK-1 to promote Wnt/β-catenin signaling activation in human fibroblasts.^38^ On the other hand, suppression of Wnt/β-catenin signaling results in attenuation of TGF-β/Smad-mediated fibrosis.^38^ Binding of transcription factors Smad4 and β-catenin/Lef1 has also been reported.^39^ Our study focused on analyzing changes at day 10 post-UUO, when we did not detect any significant changes in canonical Wnt ligands, the effector, or downstream target genes (Supplementary Fig. S4c–f). However, it does not exclude the possibility that *Lrp5* knockout affects Wnt/β-catenin signaling activation at other time points after UUO. We are aware and acknowledge that it is very challenging to exclude the involvement of Wnt/β-catenin signaling when measuring TGF-β/Smad signaling in fibrotic disease conditions, due to their reciprocal regulations and interactions. Notably, in the study of *Lrp5* deficiency in pulmonary fibrosis, the authors also acknowledged that LRP5 may play a β-catenin-independent role to regulate fibrosis, supporting our finding in the kidney.^5^ LRP6, another LDLR family member, mediates the inhibitory effect of DKK-1 through the TGF-β-JNK signaling axis in renal pericytes,^40^ suggesting a role of LRP6 independent of β-catenin. This study thus attests LRP5’s effect on promoting the fibrotic process independent of β-catenin.

LRP5 levels are upregulated in CKD, correlating with renal fibrosis. Thus, it will be very interesting to study if human patients with loss-of-function mutations of *LRP5* are less likely to develop renal fibrosis in CKD. In addition, we observed increased LRP5 levels in the diabetic glomeruli, but whether LRP5 regulates the survival and foot effacement of podocytes remains to be explored under diabetic conditions. Furthermore, LRP5 expression is detected in macrophages^41^ and monocytes,^42^ cells that play critical roles in the renal fibrogenesis.^43^ It is undetermined if LRP5 plays a role in these inflammatory cells and what signaling pathways mediate its effect. In summary, we have identified a new profibrotic role of LRP5 in driving renal fibrosis via direct interaction with TGF-β/Smad signaling, suggesting LRP5 expression as a prognostic marker for the progression of CKD.

## Materials and methods

### Animal models

Akita, *db/db*, *Lrp5*^*−/−*^ (*B6.129P2-Lrp5*^*tm1Dgen*^*/J*), and SBE-Luc mice were purchased from the Jackson Laboratory (Bar Harbor, ME). *Lrp5*^*−/−*^ mice were cross-bred with SBE-Luc mice to generate *Lrp5*^*−/−*^/SBE-Luc mice, cross-bred with OVE26 mice to generate *Lrp5*^*+/−*^/OVE26 mice. Eight-week-old mice were randomly assigned to sham surgery or UUO surgery according to an established protocol.^2^ Briefly, the left ureter was double ligated using 4-0 silk. Ureters of the sham control were manipulated without ligations. Left kidneys were collected for analyses at day 5 or day 10 post-surgery. All the procedures on mice were approved by the Institutional Animal Care and Use Committee at the University of Oklahoma Health Sciences Center (protocol number: 14-032-SSHT).

### Measurements of renal functions

Twenty-four-hour urine samples were collected. Urinary creatinine was measured by HPLC as previously described.^3^ Urinary albumin was measured using an ELISA kit for mouse albumin (Exocell, Philadelphia, PA).

### Cell culture, transfection, fractionation, and Co-IP assays

PTECs were isolated according to an established protocol.^44^ Briefly, the cortex of the kidney was dissected out carefully and chopped into small pieces. Then 1 mg/ml of collagenase solution was applied and incubated at 37 °C for 30 min, with gentle agitation. The digestion was terminated by FBS and then sequentially filtered. Fragmented proximal tubules were collected, and maintained in renal epithelial cell basal medium with a growth kit. The purity of PTECs was confirmed by immunostaining of SGLT1 (07-1417, EMD Millipore, Temecula, CA). Cells of passage 2–5 were used for experiments.

HKC-8, a human renal proximal tubule epithelial cell line (a kind gift from Dr. L. Racusen, the Johns Hopkins University, Baltimore, MD), and HEK293A cells were transfected and then cultured for 48 h before the treatments. Plasmids expressing LRP5FL, LRP5ΔN, and LRP5ΔC were generated. pcDNA3 plasmid was purchased from the Thermo Fisher Scientific (Waltham, MA), and LDLR-Flag plasmid was obtained from the Addgene (Cambridge, MA).

Cycloheximide (Sigma-Aldrich, Saint Louis, MO) treatment at 50 μM was used to measure the protein stability, with DMSO (Sigma-Aldrich, Saint Louis, MO) as control. Fractionation of cell lysates was performed according to the manufacturer’s instructions (BioVision Technology, Chester Spring, PA). For Co-IP assays, cells were washed with PBS, lysed in the IP buffer,^45^ and then proteins were pulled down using anti-Flag resin (Thermo Fisher Scientific, Waltham, MA), Ni-NTA resin (Thermo Fisher Scientific, Waltham, MA), and TβRII antibody (sc-17792, Santa Cruz, Dallas, TX). Precipitated proteins were then subjected to Western blot analysis.

### Real-time PCR

Real-time PCR was performed as described previously.^3^ Sequences of primer pairs for genes were listed in Table S1 or referred to a documented study.^12^

### Western blot analysis and ELISA

Western blot analysis was performed as previously described.^46^ Antibodies used for Western blot analysis were listed in the Table S2. GAPDH, β-actin, α-tubulin, and pan-cadherin were used as loading controls. Individual band in Western blots was semi-quantified by densitometry using the AlphaView software (Alpha Innotech, Santa Clara, CA). The densitometry of target proteins was normalized to that of individual housekeeping protein. Then the normalized ratio values were averaged in the control group. The averages of other groups were divided by that of the control group, to obtain the “fold change over the control” (with control group as “1”). TGF-β1 ELISA was performed according to the manufacturer’s instruction (R&D Systems, Minneapolis, MN).

### Luciferase activities measurement

Measurement of luciferase activity in cell lysates or kidney homogenates was performed according to the manufacturer’s instructions (Promega, Madison, WI).

### Immunohistochemistry, immunofluorescence, picro-sirius red, X-gal staining, and PAS

Kidney cryosections were used for X-gal staining (Sigma-Aldrich, Saint Louis, MO) as previously described,^45^ or immunostaining of Smad2/3 (sc-8332, Santa Cruz, Dallas, TX). Paraffin-embedded kidney sections were used for picro-sirius red staining (Roche Life Science, Indianapolis, IN), PAS (Promega, Madison, WI), immunohistochemical staining of LRP5 (ab38311, Abcam, Cambridge, MA) and collagen III (NBP2-15946, BD Pharmingen, San Jose, CA), and immunofluorescence staining of α-SMA (sc-32251, Santa Cruz, Dallas, TX), E-cadherin (610182, BD Biosciences, San Jose, CA), TβRI (NBP1-01037, Novus, Littleton, CO), SGLT-2 (ab37296, Abcam, Cambridge, MA) and LRP5 (ab38311, Abcam, Cambridge, MA). HKC-8 cells were fixed, penetrated or non-penetrated, and used for LRP5 staining (sc-390267, Santa Cruz, Dallas, TX) and Smad2/3 (sc-8332, Santa Cruz, Dallas, TX).

### Internalization of membrane receptors

HKC-8 cells were co-transfected with a control plasmid pcDNA3 or a plasmid expressing LRP5 together with a plasmid expressing either TβRI or TβRII, and cultured for 48 h. Then cells were incubated with 10 μg/ml primary antibodies for LRP5 (sc-390267, Santa Cruz, Dallas, TX), TβRI (sc-398, Santa Cruz, Dallas, TX), and TβRII (sc-17792, Santa Cruz, Dallas, TX) at 4 °C for 1 h, and washed three times with ice-cold PBS. Cells were then incubated in pre-warmed medium containing 2 ng/ml TGF-β1 at 37 °C for 7.5 min to stimulate the internalization. Non-internalized primary antibodies were removed with 0.2 M glycine buffer (pH 2.5). Then the cells were fixed with 4% paraformaldehyde and permeabilized with 0.1% Brij98 in PBS, and blocked with 20% goat serum in PBS. Internalized primary antibodies were detected with Alexa-488 conjugated goat anti-rabbit or anti-mouse secondary antibodies (Thermo Fisher Scientific, Waltham, MA).

### Statistical analysis

Experiments were performed at least three times, and representative data was presented. All values were expressed as mean ± SEM. Statistical comparisons among groups were performed using two-way ANOVA with pair-wise multiple comparisons or unpaired Student’s *t*-test. Statistical significance was set at *p* < 0.05.

## Supplementary information

Certificate of English Editing

Suppl. Figs
