# Supplementary material for: A novel role of LRP5 in tubulointerstitial fibrosis through activating TGF-β/Smad signaling
Source: Signal Transduct Target Ther. 2020 Apr 29;5:45. doi: 10.1038/s41392-020-0142-x (PMC7188863; doi:10.1038/s41392-020-0142-x)
Supplement: Supplementary file 1 — Certificate of English Editing [file 41392_2020_142_MOESM1_ESM.pdf]

Feb/08/2020

Guangzhou Liheng Translation Consulting Co. Ltd
1028-A03, Taojin Business Center, 98# Hengfu Rd., Yuexiu District Guangzhou, China. 510000
Tel: +86-20-83582259
E-mail: med-trans-4u@hotmail.com

Certificate

To whom it may concern,

This document certifies that the paper "LRP5 drives tubulointerstitial fibrosis in chronic kidney diseases through activating TGF-β/Smad signaling" has been professionally edited by Gayle Cadzow, a professional English language science editor specializing in the revision of life science papers. The quality of the edit is guaranteed to be clear and free of errors.

Best regards,

Yushan Cai, Project Manager

Guangzhou Liheng Translation Consulting Co. Ltd
