# Supplementary material for: A novel role of LRP5 in tubulointerstitial fibrosis through activating TGF-β/Smad signaling
Source: Signal Transduct Target Ther. 2020 Apr 29;5:45. doi: 10.1038/s41392-020-0142-x (PMC7188863; doi:10.1038/s41392-020-0142-x)
Supplement: Supplementary file 2 — Suppl. Figs [file 41392_2020_142_MOESM2_ESM.docx]

**Supplementary Materials for**

**A novel role of LRP5 in tubulointerstitial fibrosis through activating TGF-β/Smad signaling**

Xuemin He^1, 2^, Rui Cheng^2^, Chao Huang^2^, Yusuke Takahashi^2,^ ^3^, Yanhui Yang^2, 4^, Siribhinya Benyajati^2^, Yanming Chen^1^, Xin Zhang^2^, Jian-xing Ma^2,3†^

^1^ Department of Endocrinology Metabolism Diseases, The Third Affiliated Hospital of Sun Yat-Sen University, Guangzhou, Guangdong 510630, China

^2^ Department of Physiology, College of Medicine, University of Oklahoma Health Sciences Center, Oklahoma City, Oklahoma 73104, U.S.A.

^3^ Department of Medicine, Section of Endocrinology and Diabetes, [Harold Hamm Diabetes Center](http://haroldhammdiabetes.com/), University of Oklahoma Health Sciences Center, Oklahoma City, Oklahoma 73104, U.S.A.

^4^ NHC Key Laboratory of Hormones and Development (Tianjin Medical University), Tianjin Key Laboratory of Metabolic Diseases, Tianjin Medical University Chu Hsien-I Memorial Hospital & Tianjin Institute of Endocrinology, Tianjin 300134, China

^†^ Correspondence: Jian-xing Ma, M.D., Ph.D.
Address: 941 Stanton L. Young Blvd., BSEB 328B, Oklahoma City, OK 73104, U.S.A.

Tel: (405) 271-4372; Fax: (405) 271-3973

E-mail: [jian-xing-ma@ouhsc.edu](mailto:jian-xing-ma@ouhsc.edu)

**This PDF file includes:**

Figures. S1 to S9

Tables S1 to S2

**
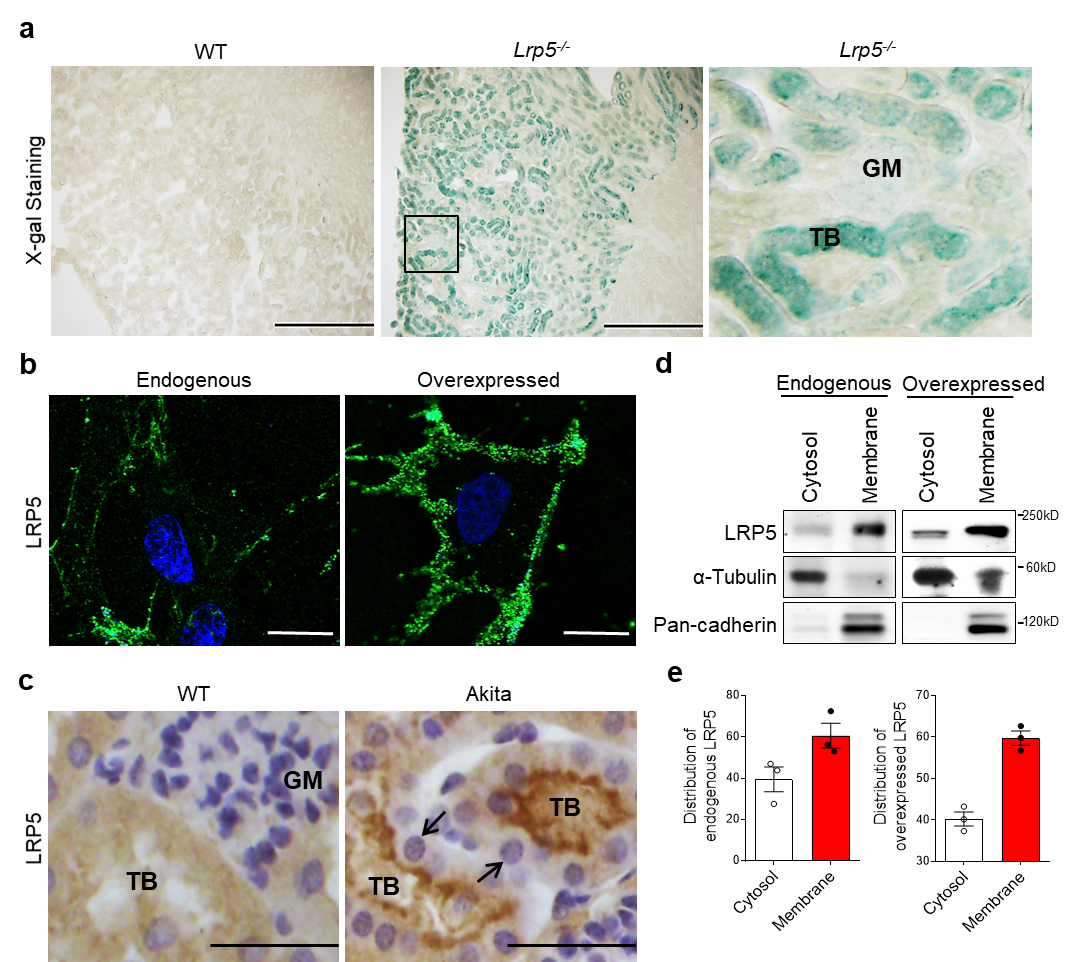
**

**Fig. S1. Localization of LRP5 in the kidney and renal tubule epithelial cells.** (**a**) X-gal staining of kidney cryosections from 8-week-old WT and *Lrp5^LacZ/LacZ^* mice under normal conditions. The *LacZ* reporter gene is knocked in the *Lrp5* locus. The right panel is the enlarged image of the boxed area in the middle panel (scale bar=500 μm). (**b**) Immunostaining of membrane LRP5 in HKC-8 cells without/with LRP5 overexpression (scale bar=10 μm). (**c**) Representative images of LRP5 immunohistochemistry from the kidneys of 3-month-old WT and Akita mice. Black arrows indicate polarized distribution of LRP5 to the apical membrane of renal tubules (scale bar=20 μm). (**d**) Western blot analyses and (**f**) densitometry quantification of LRP5 in normal HKC-8 cells (left panel) and LRP5-overexpressing HKC-8 cells (right panel) (n=3). All values are expressed as mean±SEM. TB: tubule; GM: glomerulus.

**
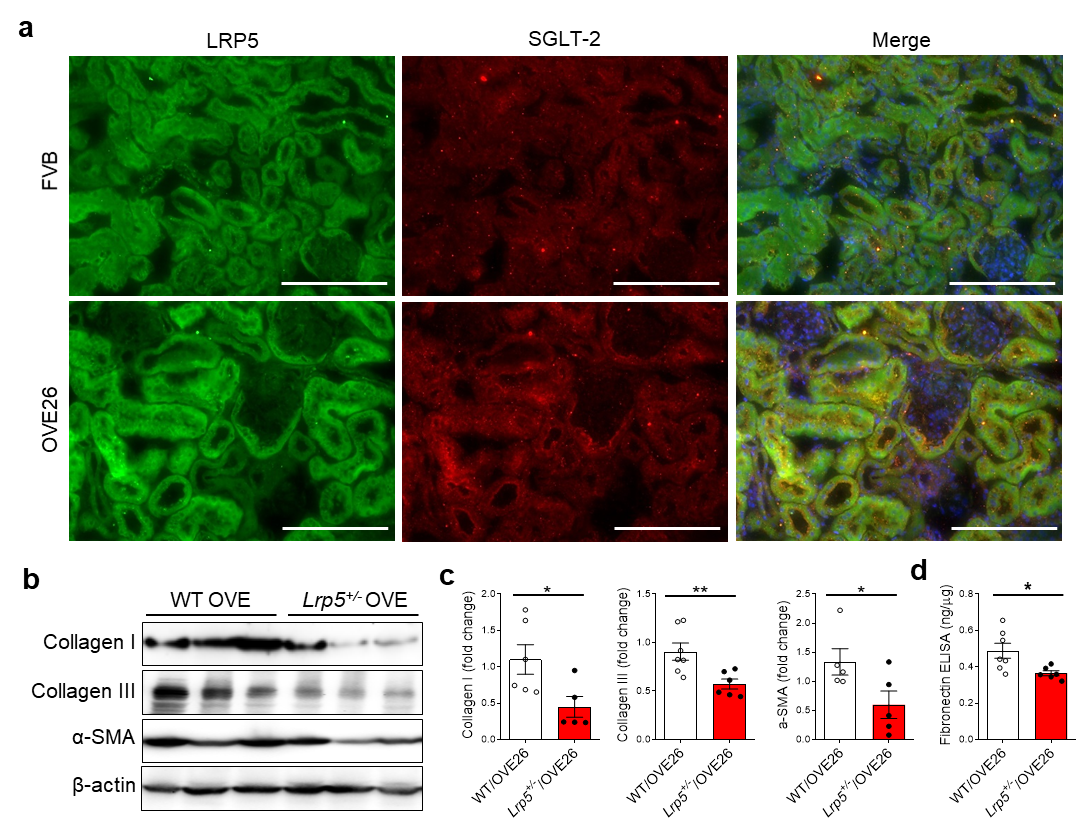
**

**Fig. S2. Knockout of *Lrp5* alleviates the overexpression of fibrotic factors in the kidney of a diabetic nephropathy model.** (**a**) Immunostaining of LRP5 (green color; scale bar=25 μm) and SGLT-2 (red color; scale bar=25 μm) in the kidney sections from 6-month-old FVB and OVE26 mice. (**b**) Western blot analyses and (**c**) densitometry quantification of collagen I, collagen III, and α-SMA in kidney homogenates from 6-month-old WT/OVE26 and *Lrp5^-/-^*/OVE26 mice (n=5-7). (**d**) ELISA of fibronectin in the kidney homogenates from 6-month-old WT/OVE26 and *Lrp5^-/-^*/OVE26 mice (n=7-8). All values are expressed as mean±SEM. ^*^*p*<0.05; ^**^*p*<0.01, by unpaired Student’s *t* test or two-way ANOVA with pair-wise multiple comparisons.

**
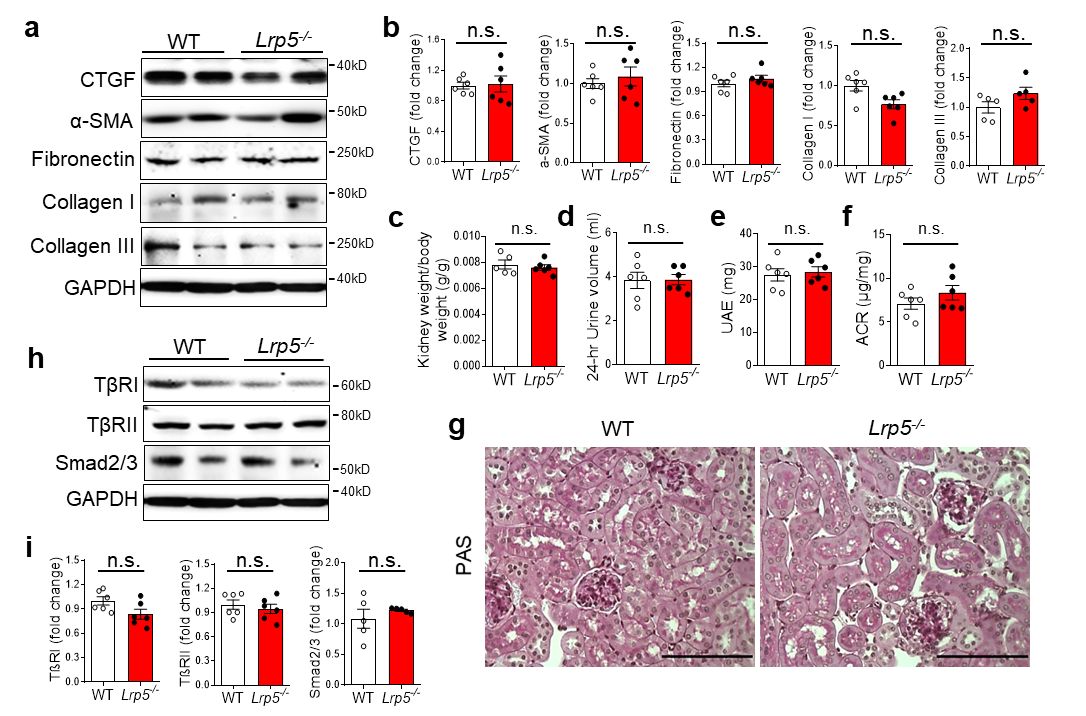
**

**Fig. S3. The renal structure and functions, levels of fibrosis factors and TGF-β signaling in *Lrp5^-/-^* kidneys were not altered under normal conditions.** (**a, h**) Western blot analyses and (**b, i**) densitometry quantification of (**a, b**) fibrosis factors CTGF, α-SMA, fibronectin, collagen I, and collagen III, and (**h, i**) TGF-β signaling components including TβRI, TβRII, and Smad2/3 in kidney homogenates from 8-week-old WT and *Lrp5*^-/-^ mice under normal condition (n=5-6). **c** The ratio of kidney weight to body weight (kidney weight/body weight), (**d)** 24-hr urine volume, 24-hr urinary albumin excretion (UAE), and (**f**) ratio of albumin to creatinine (ACR) are measured and compared between 8-week-old *Lrp5^-/-^* mice and age-matched WT controls under normal condition (n=5-6). **g** PAS of kidneys from 8-week-old *Lrp5^-/-^* mice and age-matched WT controls (scale bar=100 μm). All values are expressed as mean±SEM. n.s., not statistically significant, by unpaired Student’s *t* test.

**
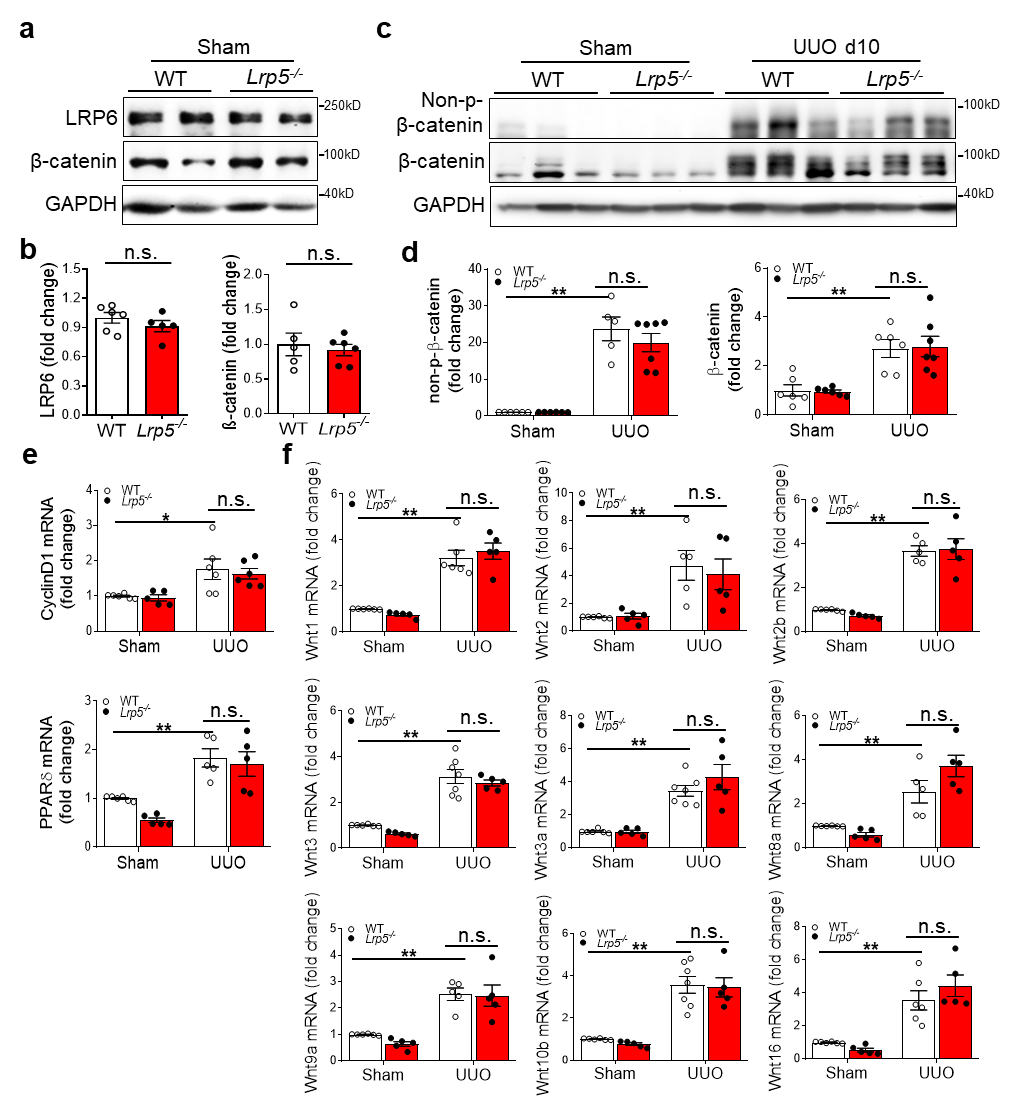
**

**Fig. S4. Knockout of *Lrp5* does not affect Wnt/β-catenin signaling.** (**a**) Western blot analyses and (**b**) densitometry quantification of the co-receptor LRP6 and effector β-catenin of Wnt/β-catenin signaling in kidney homogenates from 8-week-old WT and *Lrp5^-/-^* mice under normal conditions (n=5-6). (**c**) Western blot analyses and (**d**) densitometry quantification of non-p-β-catenin and total β-catenin in kidney homogenates from WT and *Lrp5^-/-^* mice at day 10 post-surgery (n=5-7). (**e, f**) Real-time PCR measurement of mRNA levels of the downstream Wnt target genes (**e**) CyclinD1 and PPARδ, and canonical Wnt ligands (**f**) Wnt1, Wnt2, Wnt2b, Wnt3, Wnt3a, Wnt8a, Wnt9a, Wnt10b and Wnt16 in the kidneys from WT and *Lrp5^-/-^* mice at day 10 post-surgery (n=5-7). All values are expressed as mean±SEM. ^*^*p*<0.05; ^**^*p*<0.01, n.s., not statistically significant, by unpaired Student’s *t* test or two-way ANOVA with pair-wise multiple comparisons.


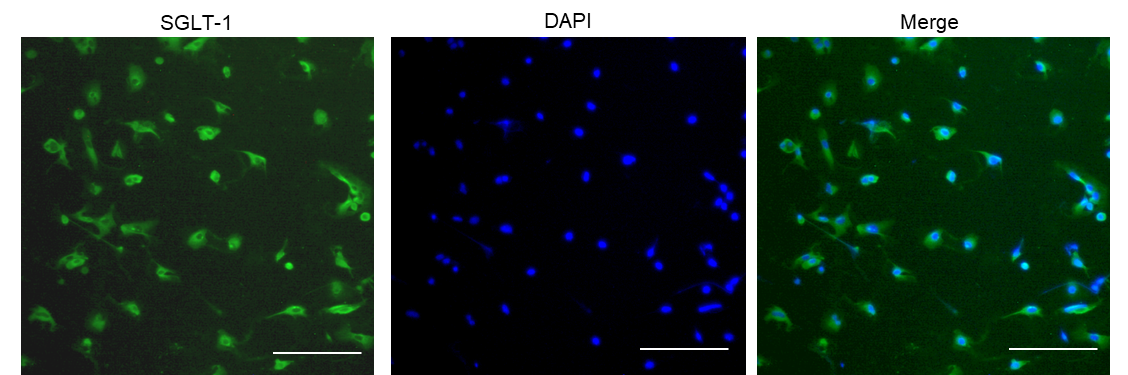


**Fig. S5. Confirmation of the purity of PTECs.** Immunostaining of SGLT1 in PTECs isolated from the kidneys of 6-week-old C57BL/6J mice (green color; scale bar=200 μm).

**
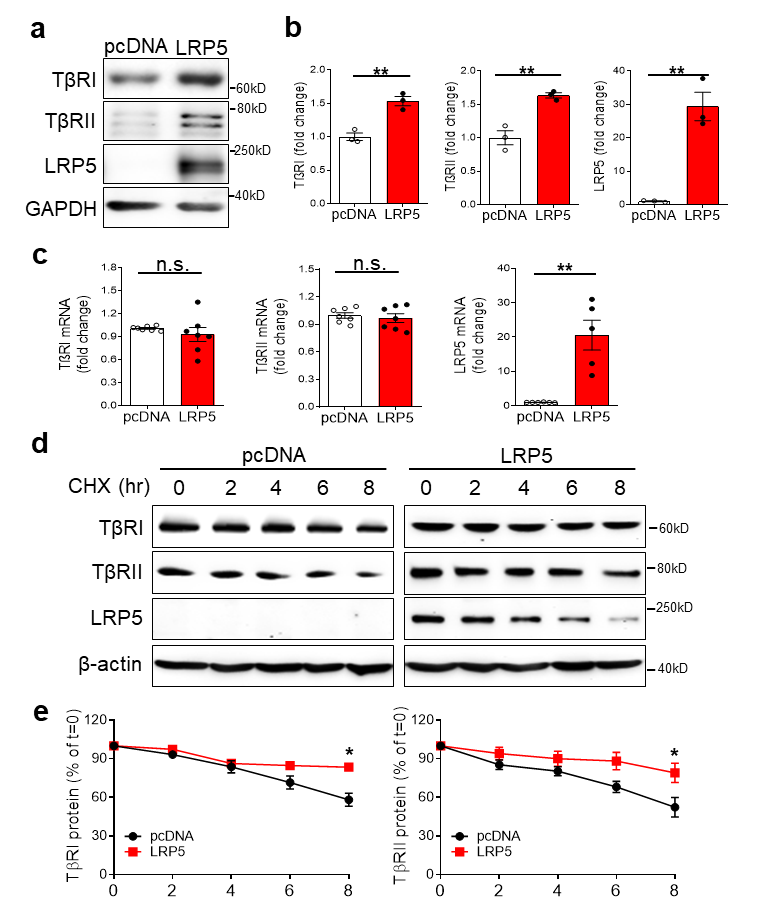
**

**Fig. S6. LRP5 stabilizes TβRs in renal tubule epithelial cells.** (**a**) Western blot analyses, (**b**) densitometry quantification and (**c**) real-time PCR measurement of TβRI, TβRII, and LRP5 in HKC-8 cells after transfection of a control plasmid (pcDNA3) or a plasmid expressing LRP5 for 48 hr (n=3-7). (**d**) Western blot analysis and (**e**) densitometry quantification of TβRI, TβRII, and LRP5 in HKC-8 cells after transfection of a pcDNA3 plasmid or a plasmid expressing LRP5 for 48 hr, followed by the treatment of 50 μM cycloheximide (CHX) for the indicated times, with DMSO as the control (n=4). All values are expressed as mean±SEM. ^*^*p*<0.05, by unpaired Student’s *t*-test.

**
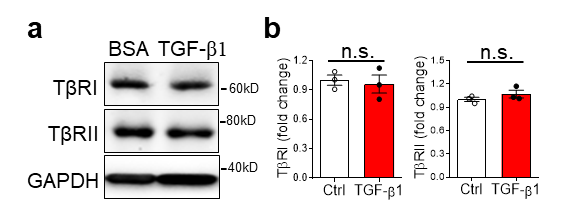
**

**Fig. S7. TGF-β1 does not increase the levels of TGF-β receptors at 30 min of the treatment.** (**a**) Western blot analysis and (**b**) densitometry quantification of TβRI and TβRII in HKC-8 cells treated with 2 ng/ml of TGF-β1 for 30 min, with bovine serum albumin (BSA) as the control (n=3). All values are expressed as mean±SEM. n.s., not statistic significant, by unpaired Student’s *t* test.

**
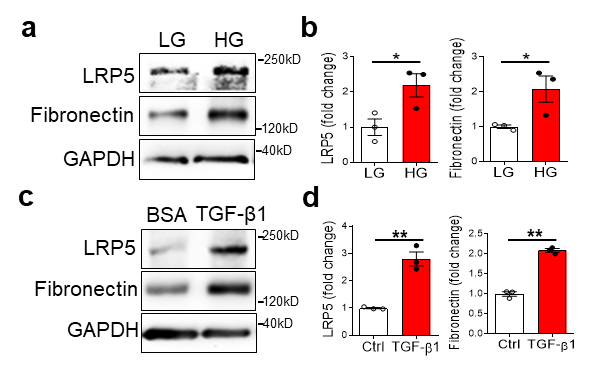
**

**Fig. S8. LRP5 levels are up-regulated by high glucose and TGF-β1 in renal tubule epithelial cells.** (**a, c**) Western blot analyses and (**b, d**) densitometry quantification of LRP5 and fibronectin in HKC-8 cells treated with (**a, b**) 30 mM D-glucose (HG) for 48 hr, with 25 mM L-glucose (LG) plus 5 mM D-glucose as the control, followed by (**c, d**) 2 ng/ml of TGF-β1 for 24 hr, with bovine serum albumin (BSA) as the control (n=3). All values are expressed as mean±SEM. ^*^*p*<0.05; ^**^*p*<0.01, by unpaired Student’s *t* test.

**
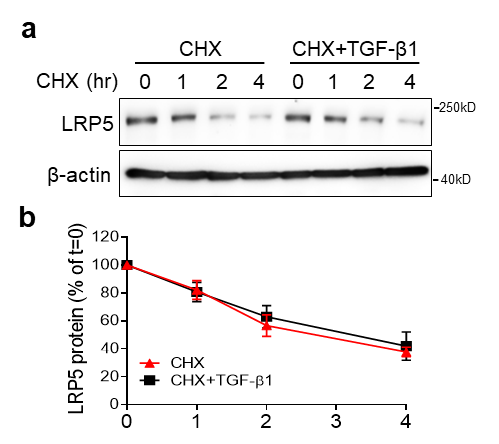
**

**Fig. S9. TGF-β1 does not prolong the half-life of LRP5.** (**a**) Western blot analysis and (**b**) densitometry quantification of LRP5 in HKC-8 cells treated with 50 μM cyclohexymide (CHX) together without/with 2 ng/ml of TGF-β1 for the indicated times, with DMSO as the control (n=3). All values are normalized to that at their individual t=0, and expressed as mean±SEM.

**Table S1. List of Real-time RT-PCR Primer Sequences**

| Mouse GAPDH forward | TTGTGATGGGTGTGAACCAC |
| --- | --- |
| Mouse GAPDH reverse | GGATGCAGGGATGATGTTCT |
| 18S forward | tgctgcagttaaaaagctcgt |
| 18S reverse | ggcctgctttgaacactctaa |
| β-actin forward | gccggcttcgcgggcgacga |
| β-actin forward | gccacacgcagctcattgtaga |
| Human LRP5 forward | CCAAGCGAGCCTTTCTACAC |
| Human LRP5 reverse | GCACGATGTCGGTGAAGTCC |
| Human TβRI forward | GCTGCTCCTCCTCGTGCT |
| Human TβRI reverse | TTGTCTTTTGTACAGAGGTGGC |
| Human TβRII forward | CTGCACATCGTCCTGTGG |
| Human TβRII reverse | GGAAACTTGACTGCACCGTT |
| Mouse PPARδ forward | GTCAAGTTCAATGCGCTGG |
| Mouse PPARδ reverse | CAGATGGAATTCTAGAGCCCG |
| Mouse CyclinD1 forward | GCGTACCCTGACACCAATCT |
| Mouse CyclinD1 reverse | CTCTTCGCACTTCTGCTCCT |

Sequences of primer pairs were obtained from the NIH qPrimerDepot website (<https://mouseprimerdepot.nci.nih.gov/>).

**Table S2. List of Antibodies for Western Blot Analyses and Co-IP Assays**

| α-SMA (sc-32251) | Santa Cruz, Dallas, TX |
| --- | --- |
| Fibronectin (sc-9068) | Santa Cruz, Dallas, TX |
| CTGF (sc-14939) | Santa Cruz, Dallas, TX |
| TGF-β1 (3711) | Cell Signaling Technology, Danvers, MA |
| Collagen III (NBP2-15946) | BD Pharmingen, San Jose, CA |
| Collagen I (ab34710) | Santa Cruz, Dallas, TX |
| E-cadherin (610182) | BD Biosciences, San Jose, CA |
| p-Smad2/3 (8828s) | Cell Signaling Technology, Danvers, MA |
| Smad2/3 (sc-8332) | Santa Cruz, Dallas, TX |
| LRP5 (D23F7) | Cell Signaling Technology, Danvers, MA |
| LRP5 (ab38331) | Abcam, Cambridge, MA |
| LRP6 | Mab2F11 |
| β-catenin (sc-7199) | Santa Cruz, Dallas, TX |
| non-p-β-catenin (D13A1) | Cell Signaling Technology, Danvers, MA |
| Mouse TβRI (NBP1-01037) | Novus, Littleton, CO |
| Mouse TβRII (NB100-91994) | Novus, Littleton, CO |
| Human TβRI (sc-9048) | Santa Cruz, Dallas, TX |
| Human TβRII (sc-400) | Santa Cruz, Dallas, TX |
| GAPDH (NB300-221) | Novus, Littleton, CO |
| β-actin (ab1801) | Abcam, Cambridge, MA |
| α-Tubulin (ab7291) | Abcam, Cambridge, MA |
| Pan-cadherin (sc-10733) | Santa Cruz, Dallas, TX |
| Flag (F 1804) | Sigma-Aldrich, St. Louis, MO |
| His-HRP (ab1187) | Abcam, Cambridge, MA |
| Biotin-HRP (4800-30-06) | R&D Systems, Minneapolis, MN |
| α-Tubulin (ab7291) | Abcam, Cambridge, MA |
| Pan-cadherin (sc-10733) | Santa Cruz, Dallas, TX |
| Flag resin | Thermo Fisher Scientific, Waltham, MA |
| Ni-NTA resin | Thermo Fisher Scientific, Waltham, MA |
| Streptavidin resin | Thermo Fisher Scientific, Waltham, MA |
| LRP5 (IP, sc-390267) | Santa Cruz, Dallas, TX |
| TβRII (IP, sc-17792) | Santa Cruz, Dallas, TX |
